# Basic Values, Career Orientations, and Career Anchors: Empirical Investigation of Relationships

**DOI:** 10.3389/fpsyg.2017.01556

**Published:** 2017-09-12

**Authors:** Marc Abessolo, Jérôme Rossier, Andreas Hirschi

**Affiliations:** ^1^Research Center in Vocational Psychology and Career Counseling, Institute of Psychology, University of Lausanne Lausanne, Switzerland; ^2^Department of Work and Organisation Psychology, Institute for Psychology, University of Bern Bern, Switzerland

**Keywords:** Schwartz's basic values, protean career orientation, boundaryless career orientation, career anchors, relationships

## Abstract

In today's dynamic and uncertain career context, values play an important role for career choice and lifelong career self-management. Values are desirable goals that are sought by individuals to satisfy their needs and are important for understanding career orientations in terms of protean and boundaryless career orientations and career anchors. However, how career orientations or career anchors fit into a well-established and supported model and into the structure of basic human values remains an important and under-investigated question. The aim of this study was to use Schwartz's model of structural values to empirically explore the relationships and structural correspondences among basic values, career orientations, and career anchors. A heterogeneous sample of 238 employees from French-speaking Switzerland (Mage = 35.60, *SD* = 13.03) completed the Portrait Values Questionnaire (PVQ5X), the Protean and Boundaryless Career Attitudes Scales (PCAS, BCAS), and the Career Orientation Inventory (COI) via an anonymous and confidential survey questionnaire. The results showed that it was possible to meaningfully position both career orientations and career anchors in Schwartz's values structure. The protean and boundaryless career orientations were positively related to Schwartz's basic values that emphasized openness to change and career anchors meaningfully followed the motivational continuum of these basic values. Overall, the overlap among the basic values, career orientations, and career anchors appeared relatively important, suggesting that these basic values, orientations, and anchors should be considered simultaneously to understand and address the factors and processes underlying individuals' career choices and paths.

## Introduction

Empirical evidences from person-organization fit (e.g., Arthur et al., 2006) suggest that individuals are more likely to choose careers in organizations that match their personal values. Inversely, a person-organization values miss-fit is likely to negatively impact individuals' job satisfaction, commitment to the organization, and intention to remain in the organization (for a review see Verquer et al., 2003). Thus, in time of uncertain career prospects including employment insecurity and economic crisis (e.g., Mucci et al., 2016), personal characteristics such as values have become essential and critical components for career choices and lifelong career self-management and are significant determinants of individuals' career development, stability, and success as well as well-being at work. However, the relationships and correspondences between personal values and career orientation constructs yet remain unclear and under-investigated.

The present paper aims to empirically explore the relationships, structural correspondences, and shared variance among basic values, career orientations, and career anchors. It is important to evaluate the overlap among these constructs to determine whether it is useful to consider these constructs simultaneously in investigations of factors and processes underlying individuals' careers choices and paths. Values are desirable goals that are pursued by individuals to satisfy their needs (Rounds and Jin, 2013); protean and boundaryless career orientations are relatively stable career preferences and attitudes (Briscoe et al., 2006); and career anchors represent individuals' inner definitions and experiences of their career needs, values, and talents (Schein, 1990). Thus, the present study aims to empirically investigate the relationships among Schwartz's basic values, protean and boundaryless career orientations, and career anchors using confirmatory factor, multinational scaling, and canonical correlation analyses techniques. We recruited a heterogeneous sample of employees to provide a more adequate and precise picture of the relationships and correspondences among these constructs to evaluate the extent to which these constructs overlap. Moreover, we advance existing knowledge regarding these constructs by investigating the degree to which these constructs capture the same latent domain (i.e., share variance). Therefore, we contribute to the career literature by providing empirical evidence regarding the strength and structure of the relationships among these constructs to facilitate future research development and applications. Finally, the present study completes meaningfully a recent work, using the same sample in respect of best recommended guidelines (cf. Kirkman and Chen, 2011), by Abessolo et al. (2017) that showed close relation among work values, as defined by Super (1970) and Dawis and Lofquist (1984), and protean and boundaryless career orientations (Briscoe et al., 2006). We herein provide a different insight and perspective by clarifying theoretically and empirically the dynamic structure of relations among Schwartz's (Schwartz et al., 2012) more recent model of basic values, protean and boundaryless career orientations, and Schein's (1990) career anchors.

### Basic values

Values are overarching and desirable goals sought by individuals to satisfy their needs (Rounds and Jin, 2013). Values serve as “guiding principles” that influence attitudes and behaviors (Schwartz, 1992). Theories of values are rooted in personality and social psychology (for a review, see Rohan, 2000). The most comprehensive theory of values was proposed by Schwartz (1992), who, in collaboration with Blisky (Schwartz and Bilsky, 1987), identified the following five common features of values in the literature: (1) concepts or beliefs (2) regarding desirable end states or behaviors (3) that transcend specific situations, (4) guide the selection or evaluation of behavior and events, and (5) are ordered by relative importance (p. 551). This understanding of values involves cognitive (e.g., beliefs), affective (e.g., desires), and behavioral (e.g., actions) components. Currently, Schwartz's (1992) theory of values remains the most comprehensive model guiding research studies investigating values (Rounds and Armstrong, 2005). Schwartz (1992) conceptualized 10 universal or basic values that fulfill the three universal biological needs of human existence, i.e., social interactions, functioning groups and the survival of groups, as follows: self-direction, stimulation, hedonism, power, achievement, security, conformity, tradition, universalism, and benevolence. The 10 basic values are organized into a circular structure of motivations (circumplex) along the following two bipolar dimensions: openness to change (including self-directed and stimulation basic values) vs. conservation (including security, tradition, and confirmation basic values) and self-enhancement (including achievement and power basic values) vs. self-transcendence (including benevolence and universalism basic values). The Schwartz Value Survey and the Portrait Values Questionnaire (SVS; PVQ; Schwartz et al., 2006, 2012) have been designed to measure these values.

### Protean and boundaryless career orientations

Protean (Hall, 2002) and boundaryless (Defillippi and Arthur, 1996) career orientations have been posited as alternative models to the traditional career model, which emphasizes long-term employment in one or two organizations, firm specific skills and training programs, and career advancement and success as measured by pay, promotion, and status (Sullivan, 1999). Changes in the global economy and organizational structures have resulted in increased uncertainty in careers (Bauman, 2007), job insecurity (Cappelli, 1999), and part-time and self-employment (Sullivan and Baruch, 2009). Thus, contemporary workers can no longer rely on their organizations to manage their careers. Instead, workers are required to manage their own careers (Fugate et al., 2004), be more flexible (Sullivan, 1999), and acquire resilience and employability (Sullivan, 1999; Baruch, 2001). Thus, protean and boundaryless career orientations emphasize that contemporary workers are more likely to choose lateral and downward organizational moves to fulfill their personal and professional needs and are more prone to follow their own desires and values rather than those of organizations (Sullivan and Baruch, 2009). Being “protean” in one's career involves the pursuit of one's personal values and career priorities and the active management of one's career by continuously learning, training and anticipating opportunities and changes in the labor market (Briscoe and Hall, 2006). Thus, a protean career is operationalized along the two dimensions of (a) value-driven and (b) self-directed career management. Being “boundaryless” involves the willingness to pursue opportunities and relationships across organizations (Briscoe et al., 2006). Thus, a boundaryless career orientation is characterized by the physical (Inkson, 2006) and psychological (Sullivan and Arthur, 2006) willingness to cross organizational boundaries in terms of both (a) organizational mobility preference and (b) boundaryless mindset (Briscoe et al., 2006).

### Career anchors

According to Schein (1990), career anchors are the significant components of an individual's career self-concept, including concerns, needs, and values, and when confronted with an important and difficult career choice, individuals will not compromise their career anchors (Coetzee and Schreuder, 2009). Career anchors reflect the concept of “internal career,” which is defined as a subjective and personal sense and definition of one's career, and contrast with the concept of “external career,” which involves the formal and objective career stages and roles defined by organizations and related institutions (Schein, 1990). Over a period of 10–12 years, Schein (1977) identified the following eight career anchors by exploring the reasons a panel of 44 alumni chose to change jobs: autonomy/independence, pure challenge, service/dedication to a cause, security/stability, life style, technical/functional competence, general managerial competence, and entrepreneurial creativity. According to Schein (1978), individuals have one dominant career anchor that expresses their core personal needs, values, and talents. However, other researchers (e.g., Feldman and Bolino, 1996; Martineau et al., 2005) have suggested the existence of multiple dominant career anchors that emerge separately from needs, values, or talents and that new career anchors might emerge from changes in contemporary workers' needs and preferences, such as the internationalism career anchor (Suutari and Taka, 2004; Lazarova et al., 2014).

### Relationships among basic values, career orientations, and career anchors

Theoretical relationships and empirical correspondences among basic values, career orientations, and career anchors have been provided in recent studies. Based on Schwartz's structural model of values and empirical findings of relationships among career anchors (cf. Igbaria et al., 1999; Petroni, 2000), Wils et al. (2010) were the first to theoretically explore the conceptual correspondence between Schein' (1990) career anchors and Schwartz's (1992) circumplex structure of basic values. Their findings showed that Schwartz' self-enhancement values were significantly related to the managerial competence and identity career anchors and that self-transcendence values were significantly related to the service/dedication to a cause and technical functional competence career anchors. In contrast, Schwartz's openness to change values were positively related to pure challenge and entrepreneurial creativity, while conservation values correlated with the security/stability and lifestyle career anchors. However, Wils et al. (2016), who reviewed existing atheoretical and theoretical models of structural relationships among career anchors (Schein, 1990; Feldman and Bolino, 1996; i.e., Bristow, 2004; Chapman, 2009), criticized this theoretical and structural model due to its lack of representativeness of Schein's career anchors. Based on the work by Wils et al. (2010, 2016) subsequently proposed a theoretical structural model that rearranged Schein's career anchors into four quadrants according to Schwartz's circumplex logic of basic values and the attached career meaning (i.e., careerist, protean, social, and bureaucratic). Using a sample of 313 graduates in the field of management sciences, Wils et al. (2016) observed that their structural model was superior to other models using a representative scale of Schein's career anchors (Schein, 1990) and a robust statistical analysis (Browne, 1992) to empirically valid the circumplex shape of the theoretical structure. Thus, their results suggested that the careerist quadrant was associated with the managerial competences career anchor; the protean quadrant was associated with the technical/functional competence, entrepreneurial creativity, pure challenge, and autonomy/independence career anchors; the social quadrant was associated with the life style and service dedication to cause career anchors; and the bureaucratic quadrant was associated with the security/stability career anchor.

Although the study by Wils et al. (2016) advanced our understanding of the correspondences among basic values, career orientations, and career anchors, limitations existed, and we aim to address these limitations in the present study. One of the limitations is that the four quadrants used by Wils and colleagues to structure the career anchors are based on a theoretical conceptualization rather than on empirical evidence. Moreover, these authors did not empirically assess career meaning (i.e., careerist, protean, social, and bureaucratic) to provide empirical evidence for the four quadrants. Finally, their study was based only on Schein's career anchors scale to test their theoretical structural model.

Considering the structure and relationships suggested by Wils et al. (2010, 2016), the aim of this project is to empirically explore the correspondence among Schwartz's basic values, protean and boundaryless career orientations, and career anchors in a heterogeneous sample of workers. Moreover, we contribute to the career research literature by elucidating theoretical suppositions of relationships among values and career related meaning and anchors. First, Schwartz's values that emphasize openness to change should be positively related to the protean and boundaryless career orientations because theoretical arguments and some empirical findings suggest that protean and boundaryless individuals are driven by the needs of freedom, growth, self-determination (Hall, 2004; Segers et al., 2008), intrinsic work values (Abessolo et al., 2017), and opportunities to learn. According to Wils et al. (2016), these values lead to the development of specific expertise or professionalism that apply to multiple organizations. Therefore, we might expect to also find positive associations with the technical/functional competence, entrepreneurial, creativity, pure challenge, autonomy/independence, and international career anchors. Second, Schwartz's values that emphasize self-enhancement should be positively associated with the managerial competences career anchor, which represents careerist individuals, who value professional success, influence, and power (Wils et al., 2016). Third, Schwartz's values that emphasize conservation should be positively related to the security/stability career anchor because this motivational domain suggests continuity, stability, and bureaucratically based relationships between individuals and organizations. Finally, Schwartz's values that emphasize self-transcendence should be positively associated with the life style and service/dedication to a cause career anchors, which involve social needs and connections. These anchors involve a greater self-awareness, awareness of others' need, helping others, and finding more meaning in one's life and society (Wils et al., 2016). Therefore, we expect to confirm the following hypotheses:

*Hypothesis 1*. Schwartz's values that emphasize openness to change will be associated with the protean and boundaryless career orientation sub-dimensions and the technical/functional competence, entrepreneurial, creativity, pure challenge, autonomy/independence, and international career anchors.

*Hypothesis 2*. Schwartz's values that emphasize self-enhancement will be associated with the managerial competences career anchor.

*Hypothesis 3*. Schwartz's values that emphasize conservation will be associated with the security/stability career anchor.

*Hypothesis 4*. Schwartz's values that emphasize self-transcendence will be associated with the life style and service/dedication to cause career anchors.

## Materials and methods

### Procedure and participants

Participants were recruited by email invitations or invitations posted on social media websites (e.g., Facebook). The survey invitation contained a brief description of the study purpose and a link to the on-line survey. A consent form was presented at the beginning of the questionnaire. Participants who provided their written informed consent were assured of their anonymity and confidentiality. Moreover, participants were informed that they would receive personalized feedback on their career profile based on their responses if desired. The response rate cannot be precisely estimated due to the sampling strategy used. However, of the 310 individuals who started the survey, 238 (77%) completed all questionnaires. Only data from participants who completed the entire survey were included in the analyses. As we were more interested in relationships among variables instead of group differences, the sample strategy and size may be sufficient and appropriate. Thus, the sample included 238 employees aged 16 to 65 years (*M*_age_ = 35.60, *SD* = 13.03) from the French-speaking region of Switzerland. Half of the participants were women (*n* = 121, 51%), and the majority were Swiss (86%). In addition, 46% of the participants were employed in the public sector, whereas 44% of the participants worked in the private sector. The remaining 10% of participants were self-employed. Two-thirds of the participants worked full-time (67%).

### Measures

#### Portrait values questionnaire

We used a validated French translation (Mrs. Pulfrey personal communication)^1^ of Schwartz's portrait values questionnaire (PVQ5X, Schwartz et al., 2012), which consisted of 51 items measuring the ten basic values of self-direction (6 items; e.g., “Being creative is important to him/her”), stimulation (3 items; e.g., “Excitement in life is important to him/her”), hedonism (3 items; e.g., “Having a good time is important to him/her”), achievement (3 items; e.g., “Being very successful is important to him/her”), power (6 items, e.g., “He/She pursues high status and power”), security (6 items; e.g., “It is important to him/her to live in secure surroundings”), tradition (3 items; e.g., “It is important to him/her to maintain traditional values or beliefs”), conformity (6 items; e.g., “Obeying all the laws is important to him/her”), benevolence (6 items; e.g., “It's very important to him/her to help the people dear to him/her”), and universalism (9 items; e.g., “Protecting society's weak and vulnerable members is important to him/her”).We used a six-point Likert scale ranging from 1 (not like me at all) to 6 (very much like me) (Schwartz et al., 2012).

#### Protean career orientation

We used a validated French translation (Stauffer et al., in press) of the protean career attitudes scale (PCAS; Briscoe et al., 2006), which consisted of 14 items that measured the self-directed career management (8 items; e.g., “I am responsible for my success or failure in my career”) and value-driven (6 items; e.g., “I navigate my own career based on my personal priorities as opposed to my employer's priorities”) dimensions of the PCO. The response format consisted of a five-point Likert scale ranging from 1 (little or no extent) to 5 (to a great extent).

#### Boundaryless career orientation

A validated French translation (Stauffer et al., in press) of the boundaryless career attitudes scale (Briscoe et al., 2006) was used, which consisted of 13 items that measured the two dimensions of boundaryless mindset (8 items; e.g., “I seek job assignments that allow me to learn something new”) and mobility preference (5 reversed items; e.g., “In my ideal career, I would work for only one organization”). The response format consisted of a five-point Likert scale ranging from 1 (little or no extent) to 5 (to a great extent).

#### Career orientation inventory

We used a validated French translation (Cerdin, 2007) of the Career Orientation Inventory (COI; Schein, 1990), which consisted of 45 items that measured the eight career anchors (5 items each; except for creativity, which had 2 items, and entrepreneurial, which had 3 items and was separated from entrepreneurial creativity) of autonomy/independence (e.g., “I will feel successful in my career only if I achieve complete autonomy and freedom”), creativity (e.g., “I am most fulfilled in my career when I have been able to build something that is entirely the result of my own ideas and efforts”), entrepreneurial (e.g., “I am always on the lookout for ideas that would permit me to start my own enterprise”), lifestyle (e.g., “I dream of a career that will permit me to integrate my personal, family, and work needs”), international (e.g., “I feel successful only if I work in an international environment”), managerial competence (e.g., “I am most fulfilled in my work when I have been able to integrate and manage the efforts of others”), pure challenge (e.g., “I will feel successful in my career only if I face and overcome very difficult challenges”), security/Stability (e.g., “I seek jobs in organizations that will give me a sense of security and stability”), service/dedication to a cause (e.g., “I am most fulfilled in my career when I have been able to use my talents in the service of others”), (Bentler and Bonett, 1980) and technical/functional competence (e.g., “I dream of being so good at what I do that my expert advice will be sought continuously”). The response format consisted of a five-point Likert scale ranging from 1 (totally disagree) to 5 (totally agree).

### Analyses

#### Confirmatory factor analysis

To evaluate the structural validity of the basic values, we conducted a confirmatory factor analyses using M*plus7* (Muthén and Muthén, 1998-2012) and the robust maximum likelihood estimator. For the factor identification, we fixed each first item to 1. Several fit-indices were used to evaluate the model fit (Bentler and Bonett, 1980; Bollen, 1989; Bentler, 1990; Hu and Bentler, 1999) as follows: the Satorra-Bentler Scaled Chi-square statistic (S-Bχ2), the root mean square error of approximation (RMSEA), the standardized root mean square residual (SRMR), the comparative fit index (CFI), and the Tucker-Lewis index (TLI). RMSEA and SRMR values <0.08, CFI and TLI values >0.90, and a chi-square per degree of freedom value equal to or below 3 are considered acceptable fit indices. We also evaluated the desirability biases using Billiet and Mcclendon's (2000) procedure, which adds a common factor to the CFA model by fixing all item loadings to 1.

#### Multidimensional scaling

We investigated the content shared by the values, career orientations, and career anchors measures by performing a multidimensional scaling analysis (MDS; Kruskal and Wish, 1978) using the SPSS 22 MDS Proxscal program with ordinal proximity transformations, Euclidian distance measures, and *Z*-score transformations of values (cf. Schwartz et al., 2012). We created a perceptual map (or a scatterplot) based on the correlation matrix (treated in term of distances), which shows the similarities (i.e., the distances) among the variables (represented by the points on the map). The closer two variables are positioned on the map, the greater the correlation between these variables. In addition, the closer a particular variable is to the center of the map, the greater the correlations among this variable and all other variables. The coefficient of alienation is a stress measure that evaluates the adequacy of the projection of the points on the map. The smaller the stress value, the better the fit between the map and the data. Stress values <0.15 are considered acceptable (Schwartz and Sagiv, 1995). The number of dimensions (i.e., axes of the scatterplot) to retain can be determined according to either a scree test (Cattell, 1966; Kruskal and Wish, 1978) or the interpretability of the map (Borg and Lingoes, 1987). A two-dimensional solution usually appears to be the most interpretable solution when mapping cognitive constructs, such as values (Schwartz, 1992; Schwartz and Sagiv, 1995).

#### Canonical correlation analysis

To assess the overlap and shared variance among values, career orientations, and career anchors and more precisely determine which values significantly impact career orientations and career anchors, we conducted canonical correlation analyses (CCAs) using the syntax provided by Nimon et al. (2010). These CCAs determine whether two sets of variables share common variance (i.e., related) or are independent (i.e., unrelated). Thus, CCAs examine the extent to which the variance in one set of variables is predicted by the other set. In the present study, the CCAs provided further information regarding the correspondences between the values and career orientations and the values and career anchors measures. Thus, the results indicate which values are significantly related to career orientations and career anchors.

## Results

### Common method bias

Because we used self-reported measures, we assessed the presence of a common method effect by performing the Harman's one-factor test, followed by a confirmatory factor analysis as a *post-hoc* test. Substantial common method bias is present when (a) one general factor accounts for the majority of the variance among the variables or (b) one general CFA model fit the data well. The results showed that none of these criteria were met in our data, rejecting the notion that a common method bias is an issue in our distinct measures of basic values, career orientations, and career anchors. Thus, one general factor did not account for a substantial amount of variance (i.e., 22.54% of the total variance), and one FCA model did not fit the data as suggested by the fit indices as follows: S-Bχ2_(230)_ = 1183.79, *p* < 0.001, χ2/df = 5.14, CFI = 0.42, RMSEA = 0.132, 90% IC (0.125, 0.139), SRMR = 0.132.

### Correlations

The means, standard deviations, bivariate correlations, and Cronbach's alpha coefficients of all measures are reported in Table 1. We found positive and significant relationships between the *self-direction value* and the protean self-directed, protean values-driven, boundaryless mindset, autonomy/independence, pure challenge, creativity, entrepreneurial, and technical/functional career anchors. The *stimulation values* were positively and significantly correlated to the protean self-directed, protean values-driven, boundaryless mindset, boundaryless mobility preference, autonomy/independence, pure challenge, creativity, entrepreneurial, internationalism, management, service/dedication to cause, and technical/functional career anchors. The *hedonism values* were positively and significantly correlated to the protean self-directed, boundaryless mindset, autonomy/independence, pure challenge, creativity, entrepreneurial, internationalism, management, and technical/functional career anchors. The *achievement values* were positively and significantly correlated to the protean self-directed, protean values-driven, boundaryless mindset, autonomy/independence, pure challenge, internationalism, and life style career anchors. The *power values* were positively and significantly correlated to the boundaryless mindset, autonomy/independence, pure challenge, creativity, entrepreneurial, internationalism, management, security/stability, and technical/functional career anchors. The *security values* were positively and significantly correlated only to the security/stability career anchor. The *tradition values* were positively and significantly correlated only to the security/stability career anchor. The *conformity values* were positively and significantly correlated to both the security/stability and service/dedication to cause career anchors. The *benevolence values* were positively and significantly correlated to the protean self-directed, protean values-drive, boundaryless mindset, life style, and service/dedication to cause career anchor. Finally, the *universalism values* were positively and significantly correlated to the protean values-driven, boundaryless mindset, life style, and service/dedication to cause career anchors. These results support H1, H2, H3, and H4.

Table 1Mean, standard deviation, bivariate correlations, and Cronbach's alpha (in parenthesis).

**Table:** 

**Variable**	***M***	***SD***	**1**	**2**	**3**	**4**	**5**	**6**	**7**	**8**	**9**	**10**
**BASIC VALUES**												
1. Self-direction	5.06	0.65	(0.78)									
2. Stimulation	4.08	1.12	0.49^***^	(0.78)								
3. Hedonism	4.26	0.89	0.35^***^	0.48^***^	(0.76)							
4. Achievement	4.88	0.87	0.34^***^	0.51^***^	0.32^***^	(0.52)						
5. Power	2.81	1.01	0.09	0.26^***^	0.51^***^	0.16*	(84)					
6. Security	4.46	0.85	0.08	−0.13	0.24^***^	0.06	0.25^***^	(0.79)				
7. Tradition	3.33	1.27	−0.18	0.07	0.28^***^	0.04	0.24^***^	0.37^***^	(0.82)			
8. Conformity	3.77	1.03	−0.11	−0.13^*^	0.05	0.03	0.02	0.46^***^	0.41^***^	(83)		
9. Benevolence	5.23	0.63	0.34^***^	0.25^***^	0.33^***^	0.35^***^	0.01	0.25^***^	0.26^***^	0.27^***^	(0.80)	
10. Universalism	4.41	0.85	0.27^***^	0.21^**^	0.00	0.25^**^	−0.29^***^	0.08	0.01	0.29^***^	0.36^***^	(0.85)
**PROTEAN AND BOUNDARYLESS CAREER ORIENTATIONS**												
11. Self-directed	3.86	00	0.47^***^	0.40^***^	0.30^***^	0.22^**^	0.11	−0.09	−0.06	−0.11	0.24^***^	0.04
12. Values-Driven	3.83	0.65	0.34^***^	0.23^***^	0.13	0.22^**^	−0.06	−0.11	−0.17^*^	−0.14^*^	0.16^*^	0.14^*^
13. Boundaryless mindset	3.58	0.80	0.38^***^	0.50^***^	0.33^***^	0.29^***^	0.15^*^	−0.10	−0.10	−0.10	0.16^*^	0.15^*^
14. Mobility preference	3.26	1.05	0.12	0.18^**^	−0.02	0.06	−0.15^*^	−0.36^***^	−0.27^***^	−0.26^***^	−0.13	0.06
**CAREER ANCHORS**												
15. Autonomy/Independence	3.44	0.78	0.43^***^	0.41^***^	0.27^***^	0.16^*^	0.20^**^	−0.11	−0.12	−0.25^***^	0.02	0.03
16. Challenge	3.18	0.80	0.32^***^	0.42^***^	0.30^***^	0.17^**^	0.26^***^	−0.09	0.01	−0.13^*^	0.11	−0.05
17. Creativity	3.08	0.91	0.28^**^	0.39^***^	0.21^**^	0.12	0.20^**^	−0.14^*^	0.02	−0.14^*^	0.05	0.01
18. Entrepreneurial	2.65	1.13	0.14^*^	0.32^**^	0.20^**^	0.05	0.23^***^	−0.04	0.01	−0.08	−0.02	−0.01
19. International	2.38	0.99	0.10	0.38^***^	0.26^*^	0.21^**^	0.19^**^	−0.14^*^	−0.07	−0.14^*^	−0.11	0.06
20. Lifestyle	3.88	0.55	0.10	0.06	−0.03	0.20^**^	−0.10	0.01	0.11	0.12	0.21^**^	0.19^**^
21. Management	2.43	0.77	0.12	0.31^***^	0.38^***^	0.07	0.49^***^	0.01	0.04	−0.04	−0.04	−0.16^*^
22. Security	3.38	0.77	−0.20^**^	−0.20^**^	0.12	0.02	0.22^**^	0.46^***^	0.30^***^	0.28^***^	0.08	−0.09
23. Service to a cause	3.42	0.76	0.10	0.19^**^	0.04	0.10	−0.17^**^	−0.03	0.08	0.17^**^	0.26^***^	0.44^***^
24. Technical/functional	3.19	0.64	0.21^**^	0.22^**^	0.27^***^	0.02	0.34^***^	0.08	0.08	0.02	−0.02	−0.07

**Table:** 

**Variable**	**11**	**12**	**13**	**14**	**15**	**16**	**17**	**18**	**19**	**20**	**21**	**22**	**23**	**24**
11. Self-directed	(0.76)													
12. Values-Driven	0.54^***^	(0.70)												
13. Boundaryless mindset	0.36^***^	0.22^**^	(0.90)											
14. Mobility preference	0.15^*^	0.12	0.24^**^	(0.87)										
15. Autonomy/Independence	0.42^***^	0.33^***^	0.28^***^	0.13^*^	(0.79)									
16. Challenge	0.35^***^	0.13^*^	0.41^***^	0.06	0.36^***^	(0.80)								
17. Creativity	0.33^***^	0.15^*^	0.25^***^	0.05	0.51^***^	0.47^***^	(0.70)							
18. Entrepreneurial	0.27^***^	0.15^*^	0.20^**^	0.10	0.51^***^	0.27^***^	0.55^***^	(0.88)						
19. International	0.13^*^	0.07	0.43^***^	0.15^*^	0.32^***^	0.33^***^	0.34^***^	0.31^***^	(0.87)					
20. Lifestyle	0.08	0.15^*^	0.03	−0.02	0.08	0.02	0.06	−0.02	−0.08	(0.58)				
21. Management	0.15^*^	−0.03	0.32^***^	−0.08	0.34^**^	0.46^***^	0.41^***^	0.46^***^	0.53^***^	−0.14^*^	(0.79)			
22. Security	−0.12	−0.15^*^	−0.17^**^	−0.51^***^	−0.27^*^	−0.05	−0.05	−0.11	−0.11	0.24^***^	0.04	(0.79)		
23. Service to a cause	0.18^**^	0.18^**^	0.21^**^	0.04	0.09	0.14^*^	0.32^***^	0.21^**^	0.18^***^	0.34^***^	0.05	0.06	(0.78)	
24. Technical/functional	0.31^***^	0.08	0.25^***^	−0.04	0.31^***^	0.53^***^	0.43^***^	0.33^***^	0.28^***^	0.16^*^	0.42^***^	0.12	0.13^*^	(0.59)

* p < 0.05;

** p < 0.01;

*** *p < 0.001*.

We found negative and significant correlations between the *protean values-driven* and security and tradition values, between the *boundaryless mobility preference* and power, security, tradition, and conformity values, between the *autonomy career anchor* and conformity values, between the *pure challenge career anchor* and conformity values, between the *creativity career anchor* and security and conformity values, between the *internationalism career anchor* and security and conformity values, between the *management career anchor* and universalism value, and finally between the *security career anchor* and self-direction and stimulation values. These results are consistent with the circumplex structure of values showing oppositions between openness to change and conservation-related variables and between self-enhancement and self-transcendence related variables.

### Confirmatory factor analyses

First, we assessed whether the 10 basic values and items in Schwartz's model fitted the data by controlling for social desirability bias using the common factor method (Billiet and Mcclendon, 2000) a robust-maximum likelihood method of estimation. The fit indices of this model confirm the structure validity and distinctness of the 10 basic values as follows: S-Bχ2_(1, 168)_ = 2142.11 *p* < 0.001, χ2/df = 1.83, CFI = 0.83, RMSEA = 0.055, 90% IC (0.051, 0.059), SRMR = 0.071.

Second, we assessed the measurement model by investigating a second-order model of Schwartz' basic values, protean career orientation, boundaryless career orientation, and career anchors as distinct but related factors with their respective sub-scales and items. The results show fit indices of S-Bχ2_(7, 102)_ = 12491.81, *p* < 0.001, χ2/df = 1.76, CFI = 0.66, RMSEA = 0.056, 90% IC (0.055,0.058), and SRMR = 0.077. Thus, the confirmatory factor analyses confirmed the factorial structure of the 10 basic values and the distinctiveness of Schwartz's basic values, protean and boundaryless career dimensions, and career anchors. However, the CFI fit index was below the acceptable threshold of 0.90, suggesting that the models were not robust to misspecification.

### Multidimensional scaling analysis

**Figure 1:**
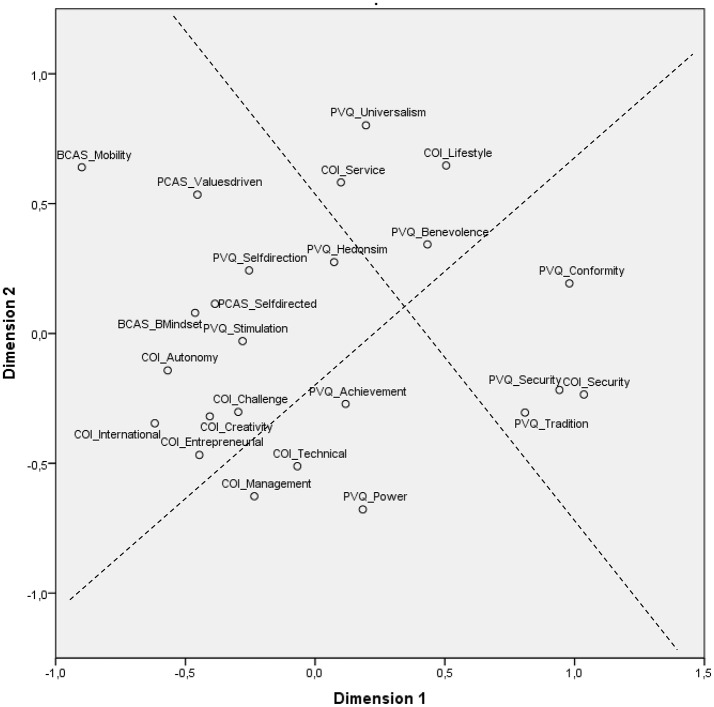
Bi-dimensional plot of basic values (PVQ), Protean (PCAS), and Boundaryless (BCAS) career orientations, and Career anchors (COI). Stress 1 = 0.15, Dispersion accounted for = 0.97, Tucker's coefficient of congruence = 0.98. The left region of the map corresponded to the *openness to chance* broad values, the lower region corresponds to the *self-enhancement* broad values, the right region corresponds to the c*onservation* broad values, and finally, the upper region corresponds to *self-transcendence* broad values.

We assessed the structure of the relationships among Schwartz's basic values, protean and boundaryless career orientations, and career anchors and evaluated whether these constructs were organized in a circumplex structure with two bipolar dimensions of openness to change, self-enhancement, conservation, and self-transcendence. As shown in Figure 1, the first MDS resulted in an acceptable coefficient of alienation of 0.15, suggesting that the projection of the constructs on the map is adequate, accurate, and interpretable. Moreover, this map appeared interesting because the four regions (subjectively drawn) showed the same compatibilities and oppositions of basic values that are found in Schwartz's theory of values. Thus, self-direction and stimulation were located in the openness to chance broad value; achievement and power were located in the self-enhancement broad value; security, tradition, and conformity were located in the conservation broad value; and benevolence and universalism were located in the self-transcendence broad value. However, hedonism differed from the theoretical order (moved between the openness to chance and self-transcendence broad values). Regarding the protean and boundaryless career dimensions and career anchors, the results showed that the four protean and boundaryless career sub-dimensions as well as autonomy/independence, international, creativity, entrepreneurial, and pure challenge career anchors were located in the openness to change broad value. The managerial competence and technical/functional competence career anchors were located in the self-enhancement broad value. The security/stability career anchor was located in the conservation broad value. Finally, the lifestyle and service/dedication to a cause career anchors were located in the self-transcendence broad value. These results support H1, H2, H3, and H4. However, surprisingly, the technical/functional competence career anchor was clearly a part of self-enhancement broad value rather than a part of the openness to chance broad value.

### Canonical correlation analyses

We examined the shared variance between two set of variables as follows: a set of Schwartz's basic value variables and a set of protean and boundaryless career dimensions and career anchor variables. Levine (1997) suggested to examine the canonical loadings (with absolute values of 0.30 and higher) to identify the variables that are strongly related to their canonical variate when the canonical correlation is significant. Thus, the canonical correlation analyses (CCAs, Table 2) revealed four significant canonical variates. The first variate (*R*c = 0.74, *R*c2 = 0.55) reflected strong positive relationships between pairs of variables of self-direction, stimulation, hedonism, and achievement basic values and protean self-directed and value-driven, boundaryless mindset and mobility preference, and autonomy, pure challenge, creativity, entrepreneurial, international, management, and technical career anchors. In contrast, this first variate showed strong positive relationships between the pairs of variables of security and conformity basic values and security career anchor. Thus, these two pairs of variables were opposed, suggesting that they represent opposite sets of variables. The second variate (*R*c = 0.67, *R*c2 = 0.45) showed strong relationships between the pairs of variables of achievement, power, and security basic values and management and security career anchors. In contrast, the second variate reflected strong positive relationships between the pairs of variables of universalism basic value and boundaryless mobility preference and life style and service/dedication to a cause career anchors. Thus, when used simultaneously, both pairs of variables would be contradictory. The third variate (*R*c = 0.49, *R*c2 = 0.24) suggested that the variables of self-direction, achievement, security, tradition, conformity, benevolence, and universalism broad values were positively associated with the variables protean values-driven, life style, security, and service/dedication to a cause career anchors and negatively associated with the variables of boundaryless mobility preference and international career anchor. Finally, the fourth significant variate (*R*c = 0.42, *R*c2 = 0.18) showed strong positive relationships when used simultaneously between the pairs of variables of stimulation, achievement, conformity, and universalism basic values and boundaryless mindset and entrepreneurial, international, management, and service/dedication to cause career anchors.

**Table 2:** Canonical correlations and loadings between a set of Schwartz's basic value variables and a set of protean and boundaryless career dimensions and career anchor variables.

	**Variate 1**	**Variate 2**	**Variate 3**	**Variate 4**
Canonical correlation	0.74	0.67	0.49	0.42
Wilks's λ	0.10	0.23	0.43	0.56
χ2 (df)	507.85 (140)^***^	327.85 (117)^***^	191.88 (96)^***^	130.79 (77)^***^
**CANONICAL LOADINGS (STRUCTURE COEFFICIENTS)**				
Basic values				
Self-direction	−**0.73**	0.04	−**0.43**	0.25
Stimulation	−**0.81**	−0.02	−0.20	−**0.39**
Hedonism	−**0.38**	0.09	−0.26	−0.23
Achievement	−**0.44**	−**0.47**	−**0.33**	−**0.37**
Power	−0.22	−**0.84**	−0.09	−0.19
Security	**0.39**	−**0.44**	−**0.63**	−0.12
Tradition	0.28	−0.20	−**0.46**	−0.16
Conformity	**0.42**	−0.03	−**0.54**	−**0.31**
Benevolence	−0.15	0.21	−**0.85**	0.02
Universalism	−0.01	**0.61**	−**0.37**	−**0.53**
**PROTEAN AND BOUNDARYLESS CAREER DIMENSIONS AND CAREER**				
**ANCHORS**				
Self-directed	−**0.46**	0.21	−0.17	0.12
Values-driven	−**0.65**	−0.06	−**0.44**	0.14
Boundaryless mindset	−**0.69**	−0.02	−0.21	−**0.39**
Mobility preference	−**0.41**	**0.30**	**0.52**	−0.05
Autonomy	−**0.70**	−0.16	−0.01	0.01
Challenge	−**0.61**	−0.23	−0.15	−0.05
Creativity	−**0.55**	−0.11	−0.05	−0.10
Entrepreneurial	−**0.38**	−0.24	0.03	−**0.33**
International	−**0.45**	−0.13	**0.31**	−**0.68**
Lifestyle	−0.02	**0.30**	−**0.39**	−0.01
Management	−**0.40**	−**0.63**	0.05	−**0.38**
Security	**0.49**	−**0.46**	−**0.47**	−0.19
Service to a cause	−0.10	**0.48**	−**0.45**	−**0.58**
Technical/functional	−**0.31**	−**0.45**	−0.14	−0.14

^*^ p < 0.05;

^**^ p < 0.01;

*** *p < 0.001*.

*In bold canonical loadings with positive and negative values of 0.30 and higher. This indicates the salience of the loadings to the variates*.

In summary, first, the present study shows evidence of significant bivariate associations among basic values, protean and boundaryless career orientations, and career anchors. Second, a confirmatory factor analysis provided evidence of the validity and distinctiveness of these constructs. Third, a multidimensional scaling analysis showed the structural relationships and correspondences in reference to the circumplex logic of Schwartz's basis values. Fourth, canonical correlation analyses showed the degree to which these constructs captured the same latent domain. Therefore, the combined results provided evidence regarding the degree of shared variance among basic values, protean and boundaryless career orientations, and career anchors.

## Discussion

The aim of the present study was to empirically explore the similarities, correspondences, and shared variance among basic values, career orientations, and career anchors. More precisely, we sought to evaluate the overlap among these constructs to better understand individuals' careers motivations and paths. Overall, our results, using a heterogeneous sample of employees, confirmed our hypotheses, suggesting significant overlaps and structural correspondences among these constructs. Thus, the protean and boundaryless career orientations were positively and significantly related to Schwartz's values that emphasize openness to change. The career anchors followed meaningfully the motivational continuum of basic values according to previous research studies. We, therefore, suggest integrating these constructs to build a common understanding and framework to explore and address more precisely the relationships, factors, and processes underlying individuals' career motivations and paths.

### An integrative understanding of basic values, career orientations, and career anchors

Our research suggests that it is possible to use Schwartz's broad values to situate, organize, and structure career orientations and career anchors. Consistently with other recent studies (Wils et al., 2010, 2016), our findings show that Schwartz's values emphasizing openness to change share similarities and a common variance with the autonomy/independence, international, entrepreneurial, creativity, and pure challenge career anchors as well as the protean and boundaryless career sub-dimensions. These findings suggest a need to consider these career attitudes and anchors simultaneously as components of career-related motivations to capture individuals' feeling and readiness for change (Schwartz et al., 2006). Thus, according to Schwartz et al.'s (2006) values and principles that organize the structure of values, individuals who portray these attitudes and anchors are likely to value and pursue autonomy, stimulation, and hedonism in their careers and are likely to reject security, conformity, and tradition values. Moreover, these individuals are likely to be regulated primarily by their self-interest rather than those of others, to cope more easily with anxiety, and to focus on promotions, gains, and growth. In contrast, our findings show similarities between Schwartz's values that emphasizes conservation (i.e., security, conformity, and tradition) and the stability/security career anchor. Unlike the opposed to openness and change motivational values, individuals anchored in security would be more focused on and affected by others, experience more anxiety when confronted with uncertainty, and be more likely to prevent professional loss and threats by complying with rules and norms.

Another opposition exists between Schwartz's values that emphasize self-enhancement and those that emphasize self-transcendence. Our results show that the former shared similarities and variance with managerial competence and technical/functional competence career anchors, suggesting that individuals who are preferably anchored in the management career anchor and technical functional career anchor are more likely to express their personal interest and pursue values of power and achievement and would rather prefer to work in well-structured and organized environments that leave little room for uncertainty. However, our results show that those who endorse the self-transcendent motivational values of universalism and benevolence expressed the lifestyle and service/dedication to cause career anchors. Thus, these individuals are more likely to be opposed to the expression of selfish interests and pursue goals to grow (Schwartz et al., 2006).

Altogether, our findings contribute to the career research literature and provide a better understanding of the motivations underlying individuals' career choices and paths. Moreover, the relationships among basic values, career orientations, and career anchors were highly significant in the direction of the two bipolar dimensions and motivational continuum proposed in Schwartz' structural values model. We may conceptualize that career anchors are driven by personal values that develop in terms of career goals and reflect career orientations. However, further investigations are needed to confirm these findings and the structural correspondences. Moreover, future research studies should investigate and clarify the relationships between work values and career anchors using the well-established taxonomies of work values proposed by Super (1970) and Dawis and Lofquist (1984) and the COI Schein (1990). These studies will provide stronger empirical evidence to build a comprehensive and integrative framework of work values, career orientations, and career anchors.

The present research findings may have important practical implications for individuals. Knowledge regarding the structure of basic values, career orientations, and career anchors and their relationships may help individuals better evaluate whether their personal life values and goals correspond to the traditional/organizational (linear career) or contemporary (non-linear or protean and boundaryless careers) career paths. For instance, an individual anchored in security/stability needs may consider work environments that preferably attach importance to preserving social and professional arrangements to ensure continuity and stability. Consequently, this individual would be more satisfied with organizational career arrangements and rewards.

### Limitations and future research

The present research has some limitations. First, we used self-reported measures, which are not free of bias, such as social desirability or the common method effect. The use of different methods and approaches may provide more robust and unbiased correspondences. Second, we used a cross-sectional design, which does not provide information regarding stability and change in the relationships over time. Finally, we used a sample that is specific to a particular culture, which may limit the generalizability of these relationships and correspondences to other cultures.

Despite these limitations, the present study advances existing knowledge on the correspondences among basic values, career orientations, and career anchors. Our results show an important overlap and shared variance among these constructs, which opens avenues for future research to build a common framework and further investigate the common underlying dimensions.

### Implications for managers

According to Hall and Yip (2014) “when climate and careers are aligned, organizations and individual members perform more effectively” (p. 230). Our results provide valuable knowledge for managers to better address and align adequately employees' values to career development and success. First, we suggest managers pay attention to both employees' working climate (Hall and Yip, 2014) and perceived organizational support (Rhoades and Eisenberger, 2002). Hence, managers should provide employees with clear performance standards and reward system as well as adequate and continuous support (i.e., social or material) in line with their career needs and values. Second, we suggest managers to treat and use basic values in an integrative way in relation to career orientations and career anchors. As our results show that basic values are motivationally associated with career orientations, or career anchors, it is important to enable employees to clarify their own career value preferences and to increase their knowledge about available career options and situations. Moreover, interventions (e.g., workshops) and tools (e.g., online job platforms) could be derived that help align employees' basic values to appropriate career development opportunities.

## Conclusion

When choosing a career, taking into account personal basic values is a critical component for a satisfying and congruent career choice. We herein contribute to the career and organizational literatures by providing an integrative understanding and new empirical knowledge of the relations among basic values, career orientations, and career anchors. On the basis of the present study, we suggest to consider these constructs simultaneously to better understand and promote individuals' career choices and lifelong career self-management.

## Ethics statement

This study was carried out in accordance to the APA ethical guidelines, that is, all participants were informed of their right to leave the survey at any time without any prejudice and guaranteed anonymity and confidentiality. All participant gave their informed consent in accordance with the Declaration of Helsinki.

## Author contributions

The order of authorship corresponds to the authors' relative contributions to the substantial, direct and intellectual effort reported in the paper, and all the authors approved it for publication.

### Conflict of interest statement

The authors declare that the research was conducted in the absence of any commercial or financial relationships that could be construed as a potential conflict of interest.

## Footnotes

^1^Personal communication between Abessolo and Mrs. Pulfrey in September 2014.
