# Corrigendum: Basic Values, Career Orientations, and Career Anchors: Empirical Investigation of Relationships

**DOI:** 10.3389/fpsyg.2018.01501

**Published:** 2018-08-21

**Authors:** Marc Abessolo, Jérôme Rossier, Andreas Hirschi

**Affiliations:** ^1^Research Center in Vocational Psychology and Career Counseling, Institute of Psychology, University of Lausanne, Lausanne, Switzerland; ^2^Department of Work and Organization Psychology, Institute for Psychology, University of Bern, Bern, Switzerland

**Keywords:** Schwartz's basic values, protean career orientation, boundaryless career orientation, career anchors, relationships

In the original article, due to an unfortunate miswording, it is possible for readers to think that the PVQ5X was translated by Abessolo and colleagues, which is not the case. Therefore, and at the request of Mrs. Pulfrey, we would like to replace the citation of Abessolo et al., 2017b with a citation of a personal communication between Abessolo and Mrs. Pulfrey in September 2014. A correction has been made to Materials and Methods, sub-section Measures, sub-subsection Portrait Values Questionnaire, and the reference above has been removed.

We used a validated French translation (Mrs. Pulfrey personal communication) of Schwartz's portrait values questionnaire (PVQ5X, Schwartz et al., 2012), which consisted of 51 items measuring the ten basic values of self-direction (6 items; e.g., “Being creative is important to him/her”), stimulation (3 items; e.g., “Excitement in life is important to him/her”), hedonism (3 items; e.g., “Having a good time is important to him/her”), achievement (3 items; e.g., “Being very successful is important to him/her”), power (6 items, e.g., “He/She pursues high status and power”), security (6 items; e.g., “It is important to him/her to live in secure surroundings”), tradition (3 items; e.g., “It is important to him/her to maintain traditional values or beliefs”), conformity (6 items; e.g., “Obeying all the laws is important to him/her”), benevolence (6 items; e.g., “It's very important to him/her to help the people dear to him/her”), and universalism (9 items; e.g., “Protecting society's weak and vulnerablemembers is important to him/her”). We used a six-point Likert scale ranging from 1 (not like me at all) to 6 (very much like me) (Schwartz et al., 2012).

The authors apologize for this error and state that it does not affect the scientific conclusions of the article in any way.

The original article has been updated.

## Conflict of interest statement

The authors declare that the research was conducted in the absence of any commercial or financial relationships that could be construed as a potential conflict of interest.
